# Fracture rate in patients with myasthenia gravis: the general practice research database

**DOI:** 10.1007/s00198-012-1970-5

**Published:** 2012-04-25

**Authors:** S. Pouwels, A. de Boer, M. K. Javaid, D. Hilton-Jones, J. Verschuuren, C. Cooper, H. G. Leufkens, F. de Vries

**Affiliations:** 1 Utrecht Institute for Pharmaceutical Sciences, Universiteit Utrecht, Utrecht, the Netherlands; 2 Oxford NIHR Musculoskeletal Biomedical Research Unit, Department of Orthopaedics, Rheumatology and Musculoskeletal Sciences, University of Oxford, Oxford, UK; 3 Department of Clinical Neurology, University of Oxford, Oxford, UK; 4 Department of Neurology, Leiden University Medical Centre, Leiden, the Netherlands; 5 MRC Lifecourse Epidemiology Unit, University of Southampton, Southampton, UK; 6 Department of Clinical Pharmacy & Toxicology, Maastricht University Medical Centre, Maastricht, the Netherlands; 7 Universiteitsweg 99, 3584 CG Utrecht, the Netherlands

**Keywords:** Corticosteroids, Epidemiology, Fracture, Myasthenia gravis, Osteoporosis

## Abstract

**Summary:**

The aim of this study was to evaluate fracture risk after onset of myasthenia gravis using the UK General Practice Research Database. Overall fracture risk is not statistically increased compared with age- and gender-matched controls irrespective of glucocorticoid use, but was increased in those using antidepressants, anxiolytics or anticonvulsants.

**Introduction:**

Myasthenia gravis (MG) is a neuromuscular disease which has been associated with an increased falls risk and glucocorticoid-induced osteoporosis, recognized determinants of increased fracture risk. The aim of this study was to evaluate the risk of fracture after onset of MG.

**Methods:**

We conducted a retrospective cohort study using the UK General Practice Research Database (1987–2009). Each MG patient was matched by age, sex, calendar time and practice to up to six patients without a history of MG and we identified all fractures and those associated with osteoporosis.

**Results:**

Compared to the control cohort, there was no statistically significant increased risk observed in patients with MG for any fracture (adjusted hazard ratio [AHR] 1.11; 95 % confidence interval [CI], 0.84–1.47) or osteoporotic fractures (AHR 0.98 [95 % CI 0.67–1.41]). Further, use of oral glucocorticoids up to a cumulative dose exceeding 5 g prednisolone equivalents did not increase risk of osteoporotic fracture (AHR 0.99 [95 % CI, 0.31–3.14]) compared with MG patients without glucocorticoid exposure. However, fracture risk was higher in patients with MG prescribed antidepressants (AHR 3.27 [95 % CI, 1.63–6.55]), anxiolytics (AHR 2.18 [95 % CI, 1.04–4.57]) and anticonvulsants (AHR 6.88 [95 % CI, 2.91–16.27]).

**Conclusion:**

Overall risk of fracture in patients with MG is not statistically increased compared with age- and gender-matched controls irrespective of glucocorticoid use but was increased in those using antidepressants, anxiolytics or anticonvulsants. These findings have implications in strategies preserving bone health in patients with MG.

## Introduction

Myasthenia gravis (MG) is an automimmune disorder with symptoms of muscle weakness and fatigability, in which antibodies reduce the number of acetylcholine receptors at the post-synaptic region of the neuromuscular junction [1]. MG is relatively rare with an estimated pooled incidence rate of 5.3 per million person-years and an estimated pooled prevalence rate of 77.7 per million persons [2]. Treatment options for MG include use of cholinesterase inhibitors and immunosuppressants, including oral glucocorticoids and in selected patients plasmapheresis and thymectomy [3]. Patients with a diagnosis of MG have a normal life expectancy based on the currently available therapies [4].

MG is associated with an increased falls risk [5–7] and glucocorticoid-induced osteoporosis [8, 9]. The increased risk of falls from MG is likely to be multifactorial including severe muscle weakness [1], impaired vision as a result of ocular MG and steroid-induced myopathy [10, 11]. Recent studies in a representative sample of the total UK population have shown that treatment with glucocorticoids is associated with a substantial risk of fracture, in a wide range of chronic diseases [12, 13]. Oral glucocorticoid treatment in MG patients is regularly started with 10 mg prednisolone per day and is quickly increased towards about 60 mg per day [14, 15]. Once an effective clinical response is obtained (within about 10–12 weeks), this dose is slowly tapered down, towards 2.5–10 mg prednisolone equivalents each day or an equivalent dose on alternate days for maintenance [15]. Hence these patients are routinely exposed to significant cumulative doses of prednisolone far exceeding 1 g.

In addition to falls risk and glucocorticoid therapy, the increased risk of fracture in patients with MG may also relate to psychiatric comorbidity and its treatment. As compared with healthy patients, MG patients are more likely to have a history of central nervous system (CNS) disorders [16]. This could be the result of a central cholinergic transmission deficit, caused by blocking of acetylcholine receptors within the central nervous system [17]. Both CNS drugs such as antidepressants and antipsychotics, and the CNS diseases like epilepsy and depression have been associated with an increased risk of fracture [18–21], or osteoporosis [22, 23].

Objectives of this study are to determine the risk of fracture in patients with MG, as compared with population-based controls, and to evaluate the effects of oral glucocorticoids and CNS medication on fracture risk in patients with MG.

## Methods

### Data sources

Information for this study was obtained from the General Practice Research Database (GPRD), which comprises the computerized medical records of all patients under the care of general practitioners in the UK. Medical information on patients who are registered for medical care with a practice is supplied to the GPRD [24]. The data in GPRD have been linked to the national Hospital Episode Statistics (HES) in England, for approximately 45 % of all practices. HES includes information on the date, main discharge diagnosis and duration of hospitalisation, as provided by the NHS hospitals. Data were linked from April 2001 up to March 2007. Previous studies of GPRD data have shown a high level of data validity with respect to the reporting of fractures (>90 % of fractures were confirmed) [25, 26].

### Study population

A proxy for identifying MG patients was agreed upon by two neurologists, an expert in bone diseases and a pharmacoepidemiologist (JV, DHJ, KJ and FV). The study population consisted of all patients aged 18 years or older with at least one recorded diagnosis of MG during the period of HES or GPRD data collection (for this study, GPRD data collection started in January 1987 and ended in July 2009). Incident cases of MG were defined as individuals whose first recorded GP or hospital visit for MG was at least 1 year after their inclusion into the database. Each MG patient was matched by year of birth, sex and practice to up to six patients without a history of MG to generate a matched cohort. The index date of MG diagnosis was the date of the first record of MG after GPRD data collection had started. Each control patient was assigned the same index date as his matched MG patient. The study patients were followed up from this index date to either the end of GPRD data collection, the date of transfer of the patient out of the practice area, the patient’s death or the occurrence of fracture, whichever came first. All types of fracture were included in the analyses and classified according to the International Classification of Diseases, Tenth Revision (ICD-10) categories (HES) and corresponding read codes (GPRD). A typical osteoporotic fracture was defined as a fracture of the radius/ulna, humerus, rib, femur/hip, pelvis or vertebrae (clinically symptomatic).

Subsequently, this population was then divided into a group of probable MG cases (*n* = 834) with their matched controls and a group of possible MG cases (*n* = 232) with their matches controls. The following criteria were used to determine a probable MG case: a recording of MG in two different registries (GPRD and HES) (*n* = 205), or it has a recording of MG in at least one registry with either a letter from a neurologist confirming the patient has seen a neurologist ever before or 1 year after the diagnostic code (*n* = 291), or a record of thymectomy (*n* = 48) any time during follow-up (recorded either in GPRD or HES) or at least two prescriptions on different days of pyridostigmine, oral glucocorticoids, azathioprine, methotrexate, ciclosporin or mycophenolate mofetil any time during enrolment (*n* = 754). Possible cases were identified if they had a recording of MG in either GPRD or HES without the abovementioned prescription data, recording of thymectomy or a letter from a neurologist. Patients were excluded if they had a record of Lambert–Eaton type myasthenic syndrome, which mimics MG.

### Exposure

The indicators of MG severity selected for the study were selected from the myasthenia gravis Foundation of America postintervention status that were also recorded in the GPRD [27]. Grade 1 included patients who did not use cholinesterase inhibitors or immunosuppressants during the past 6 months. Grade 2 included patients who used immunosuppressants, but not cholinesterase inhibitors during the past 6 months. Grade 3 included patients who used pyridostigmine only during the past 6 months (and no immunosupressants), and grade 4 included patients who had been on both immunosuppressants and cholinesterase inhibitors. MG severity grade may fluctuate over time.

Potential confounders that were determined at baseline included body mass index (BMI), smoking status, alcohol status and occurrence of prior fractures. Missing data for BMI, smoking or alcohol status was treated as a separate group in the statistical models. Potential confounders that were determined for a time-dependent analysis during follow-up included age, a history of chronic diseases (including asthma/chronic obstructive pulmonary disease (COPD), rheumatoid arthritis, thyroid disorders, renal failure, cancer, congestive heart failure, cerebrovascular disease, diabetes mellitus, inflammatory bowel disease and secondary osteoporosis (based on the definition of FRAX [28]), a prescription in the 6 months before an interval for CNS medication, anti-parkinson medication, non-steroidal antiinflammatory drugs (NSAIDs), oral glucocorticoids and other immunosuppressants (azathioprine, ciclosporin, tacrolimus, mycophenolate mofetil and methotrexate). In this approach it was assumed that no residual effect was left for medication used more than 6 months before an interval. The use of oral glucocorticoids and CNS medication were stratified to average daily dose in 6 months before an interval, and use of oral glucorticoids was also stratified to cumulative dose in the year before an interval. WHO defined daily dosages were used to add up dose equivalences of various CNS medication and oral glucocorticoid substances. Within the 6 months before each interval, the average daily dose was calculated by dividing the cumulative dose by the time between the oldest prescription and the start date of the period. In addition, MG disease duration was noted, as measured from the start of follow-up.

### Statistical analysis

Time-dependent Cox proportional hazards regression was used in order to estimate hazard ratios (HRs) of fracture risk. The first analysis compared the fracture rate in MG patients with that in control patients, to yield an estimate of the HRs of fracture in MG. The second analysis examined the effect of disease severity and use of oral glucocorticoids, antidepressants, anxiolytics or anticonvulsants on fracture risk in the MG cohort.

For each analysis, the regression model was fitted with the indicators for MG severity and general risk factors. These characteristics were treated as time-dependent variables in the analysis, in which the total period of follow-up was divided into periods of 30 days, starting at the index date. At the start of each period, the presence of risk factors and indicators of MG severity were assessed by reviewing the computerized prescription and diagnosis records prior to the right censoring date. BMI, alcohol status, smoking status and occurrence of prior fracture were determined at baseline. During follow-up, the presence of a previous record for a chronic disease ever before each period of 30 days was assessed, while the presence of a medical prescription was assessed in the 6 months before each period. All characteristics, except age, were included as categorical variables in the regression models. A priori we tested for interactions between age and gender with fracture risk. Adjustments were made if any potential confounder showed a change in HR exceeding 1 %.

### Sensitivity analyses

A separate analysis was performed for probable and for possible MG patients. In a second sensitivity analysis, we excluded all patients and their matched subjects who had ever been prescribed a bisphosphonate, selective oestrogen receptor modulator, strontium ranelate or parathyroid hormone during follow-up. This in order to evaluate whether the use of bone protecting treatment had masked a true association between MG or glucocorticoid use and fracture.

## Results

Table 1 shows that there were 1,066 incident patients with probable or possible MG matched to 6,392 controls identified between 1987 and 2009. The mean age of patients with MG was 62 years and 50 % were female. Most patients with incident MG (78 %) were able to be classified with probable MG. Patients were followed for a median of 4 years.

**Table 1:** Baseline characteristics of patients with incident myasthenia gravis and control patients

	MG patients	Controls	Probable MG patients	Possible MG patients
Characteristics	(*n* = 1,066)	(*n* = 6,392)	(*n* = 834)	(*n* = 232)
Female	49.7	49.8	45.6	64.7
Mean age (years)	61.6	61.4	62.4	58.4
BMI (%)				
<20	5.2	5.5	4.3	8.2
>30	21.5	16.6	22.9	16.4
Unknown	13.0	15.5	12.6	14.7
Smoking status (%)				
Never	47.7	43.2	46.6	51.7
Current	13.8	17.6	13.5	14.7
Ex	23.2	22.0	25.5	14.7
Unknown	15.3	17.1	14.3	19.0
Alcohol status (%)				
Never	14.7	10.4	15.2	12.9
Current	57.5	59.6	57.6	57.3
Ex	5.5	3.9	6.0	3.9
Unknown	22.2	26.1	21.2	25.9
Fracture history (%)				
Any fracture	15.1	15.7	15.0	15.5
Fracture at osteoporotic sites	6.8	7.5	6.7	6.9
Hip fracture	0.8	0.6	0.8	0.4
Vertebral fracture	0.8	0.6	0.5	0.9
Radius/ulna fracture	2.8	3.9	2.6	3.4
Comorbidity ever before index date (%)				
Asthma	13.1	10.5	12.8	14.2
COPD	3.0	4.2	3.1	2.6
Congestive heart failure	2.3	2.9	2.0	3.4
Diabetes mellitus	7.9	6.9	8.8	4.7
Rheumatoid arthritis	2.6	1.3	2.8	2.2
Renal failure	1.1	0.9	1.2	0.9
Cerebrovascular disease	8.0	6.1	8.8	5.2
Inflammatory bowel disease	0.8	0.8	0.7	1.3
Cancer	18.3	18.1	18.6	17.2
Thyroid disorders	18.7	11.0	18.0	21.1
Secondary osteoporosis	6.6	4.5	6.5	6.9
Drug use in 6 months before index date (%)				
Pyridostigmine	13.0	0.0	16.5	0.4
Oral glucocorticoids	8.7	2.8	9.2	6.9
Immunosuppressants^a^	2.2	0.4	2.8	0.0
Antidepressants	10.4	8.4	10.9	8.6
Antipsychotics	1.2	1.3	1.2	1.3
Anxiolytics	8.4	5.9	7.4	12.1
Anticonvulsants	3.3	1.8	3.2	3.4
Bisphosphonates	4.1	1.8	4.2	3.9
Hormone replacement therapy	1.9	1.7	1.6	3.0

^a^Ciclosporin, azathioprine, tacrolimus, mycophenolate mofetil and methotrexate are included

When compared with their matched controls, patients with a diagnosis of MG had no increased risk of either all fractures in both unadjusted and adjusted models (adjusted hazard ratio (AHR) for any fracture 1.11 (95 % confidence interval [CI] 0.84–1.47) or typical osteoporotic fractures AHR 0.98 (95 % CI 0.67–1.41); Table 2. The fracture risk did not differ significantly among patients with probable MG (AHR for any fracture 0.89 [95% CI 0.67–1.25]; for classical osteoporotic fracture AHR 0.79 [95% CI 0.50–1.25]). In addition, no associations were observed between incident MG patients stratified by gender and by age categories.

**Table 2:** Risk of fracture in incident MG patients by type of fracture, gender and age compared to patients without MG

	Number of fractures	Rate/1,000 person-years	Age–sex adjusted HR (95 % CI)	Fully adjusted HR (95 % CI)^a^
No MG	426	12.6	1.00	1.00
MG (any fracture)	75	14.2	1.19 (0.93–1.52)	1.11 (0.84–1.47)
Fracture at osteoporotic sites	43	8.2	1.13 (0.82–1.56)	0.98 (0.67–1.41)
Hip fracture	8	1.5	0.85 (0.41–1.77)	0.61 (0.26–1.45)^b^
Vertebral fracture	9	1.7	2.85 (1.31–6.18)	2.13 (0.82–5.51)^c^
Radius/ulna fracture	11	2.1	0.92 (0.49–1.73)	1.02 (0.51–2.04)^d^
Other fracture	15	2.8	1.00 (0.58–1.71)	0.86 (0.47–1.59)^e^
Fracture at non-osteoporotic sites	32	6.1	1.29 (0.89–1.89)	1.42 (0.93–2.17)^f^
By gender^g^				
Male	27	10.5	1.11 (0.74–1.67)	0.86 (0.52–1.42)
Female	48	18.6	1.24 (0.91–1.68)	1.20 (0.86–1.69)
By age at MG diagnosis^h^				
18–39	10	12.4	1.83 (0.90–3.69)	1.76 (0.80–3.86)
40–59	10	6.5	0.68 (0.36–1.31)	0.62 (0.29–1.29)
60–69	18	14.5	1.36 (0.82–2.25)	1.42 (0.80–2.52)
70–79	25	19.5	1.29 (0.84–4.34)	1.18 (0.72–1.92)
> = 80	12	30.4	1.11 (0.60–2.05)	0.97 (0.47–2.00)

^a^Adjusted for age, gender, use of immunosuppressants, oral glucocorticoids and antidepressants in the previous 6 months, history of smoking and alcohol use

^b^Additionally adjusted for anxiolytics and antipsychotics in the previous 6 months, history of asthma and cerebrovascular disease

^c^Additionally adjusted for use of anxiolytics, NSAIDs, anti-parkinson medication in the previous 6 months, history of COPD, rheumatoid arthritis, asthma, secondary osteoporosis and BMI status but not for history of smoking

^d^Not adjusted for history of smoking

^e^Not adjusted for use of antidepressants in the previous 6 months and not for history of smoking

^f^Additionally adjusted for history of stroke in the previous year and history of hypothyroidism and secondary osteoporosis. Not adjusted for antidepressant use and not for history of alcohol use

^g^Male MG patients are compared with male controls and female MG patients with female controls

^h^MG patients in each age group are only compared with control patients in the same age group

We then examined the effect of exposure to medications well known to be associated with an increased risk of fracture (Table 3). Surprisingly, recent exposure to oral glucocorticoids did not significantly alter fracture risk within MG patients. At osteoporotic sites of incident MG patients, fracture risk yielded an AHR of 0.81 (95 % CI 0.40–1.61) compared to MG patients who did not use oral corticosteroids in the past 6 months. Furthermore, an average daily dose exceeding 15 mg prednisolone equivalents in the past 6 months (AHR 1.17 [95 % CI 0.47–2.89]) or a cumulative dose in the year prior to each interval, exceeding 5 g prednisolone equivalents (AHR 0.99 [95 % CI 0.31–3.14]) did not significantly alter osteoporotic fracture risk. In these analyses, osteoporotic fractures were reported in respectively seven and four MG patients. The interaction term between MG and oral glucocorticoids did not reach statistical significance (*p* value > 0.05) for any and for typical osteoporotic fractures (Table 4). Finally, a sensitivity analysis in which 645 MG patients without exposure to osteoporosis therapies and their 3,647 controls were left, a diagnosis of MG did not alter risk of any (AHR 1.21 [95 % CI 0.84–1.74]) or typical osteoporotic fracture (AHR 1.44 [95 % CI 0.89–2.34]).

**Table 3:** Risk of any and osteoporotic fracture among incident MG patients by drug exposure

	Risk of any fracture		Risk of fracture at osteoporotic sites	
	Number of fractures	Fully adjusted HR (95 % CI)^a^	Number of fractures	Fully adjusted HR (95 % CI)^a^
MG by use of oral glucocorticoids by cumulative dose in grams prednisolone equivalents in the previous year				
No oral glucocorticoid use	47	1.00	27	1.00
Any oral glucocorticoid use	28	0.88 (0.52–1.47)	16	0.75 (0.38–1.50)
<2.5 g prednisolone eq	13	0.80 (0.42–1.53)	7	0.63 (0.26–1.53)
2.5–5.0 g prednisolone eq	10	1.11 (0.54–2.26)	5	0.83 (0.31–2.25)
> = 5.0 g prednisolone eq	5	0.73 (0.27–1.94)	4	0.99 (0.31–3.14)
MG by history of drug use in previous 6 months				
No oral glucocorticoid use	48	1.00	28	1.00
Oral glucocorticoid use	27	0.97 (0.58–1.63)	15	0.81 (0.40–1.61)
<7.5 mg prednisolone eq/day	10	0.99 (0.49–2.03)	5	0.70 (0.26–1.92)
7.5–15 mg prednisolone eq/day	8	1.00 (0.46–2.16)	3	0.57 (0.17–1.93)
> = 15 mg prednisolone eq/day	9	0.93 (0.44–1.99)	7	1.17 (0.47–2.89)
No antidepressant use	59	1.00	31	1.00
Antidepressant use	16	2.15 (1.22–3.79)	12	3.27 (1.63–6.55)
<20 mg fluoxetine eq/day	9	1.88 (0.92–3.86)	7	2.77 (1.18–6.50)
> = 20 mg fluoxetine eq/day	7	2.61 (1.18–5.80)	5	4.32 (1.64–11.38)
No anxiolytic use	61	1.00	32	1.00
Anxiolytic use	14	1.80 (0.97–3.34)	11	2.18 (1.04–4.57)
<10 mg diazepam eq/day	10	1.72 (0.85–3.47)	8	2.10 (0.90–4.86)
> = 10 mg diazepam eq/day	4	2.07 (0.73–5.82)	3	2.41 (0.71–8.12)
No anticonvulsant use	64	1.00	36	1.00
Anticonvulsant use	11	5.36 (2.76–10.39)	7	6.88 (2.91–16.27)
<1.0 g carbamazepine eq/day	8	4.88 (2.27–10.50)	5	5.45 (2.03–14.62)
> = 1.0 g carbamazepine eq/day	3	7.10 (2.13–23.62)	2	18.18 (3.88–85.15)
No antipsychotic use	74	1.00	42	1.00
Antipsychotic use	1	1.30 (0.17–9.76)	1	1.41 (0.17–11.65)

*eq* equivalents

^a^Adjusted for the same confounders as described below Table 2 for any and osteoporotic fracture, but the confounder is not added to the model if it is similar to the drug being investigated

**Table 4:** Risk of any and osteoporotic fracture among incident MG patients and controls by drug exposure

	Risk of any fracture fully adjusted HR (95 % CI)^a^		*p* value of interaction term^b^	Risk of fracture at osteoporotic site fully adjusted HR (95 % CI)^a^		*p* value of interaction term^b^
	MG patients	Controls		MG patients	Controls	
Drug use in previous 6 months						
No oral glucocorticoid use	1.00	1.00		1.00	1.00	
Oral glucocorticoid use	0.88 (0.52–1.47)	1.50 (1.02–2.20)	0.217	0.75 (0.38–1.50)	1.86 (1.23–2.83)	0.065
No antidepressant use	1.00	1.00		1.00	1.00	
Antidepressant use	2.15 (1.22–3.79)	1.50 (1.15–1.96)	0.608	3.27 (1.63–6.55)	1.63 (1.18–2.27)	0.260
No anxiolytic use	1.00	1.00		1.00	1.00	
Anxiolytic use	1.80 (0.97–3.34)	1.14 (0.82–1.59)	0.101	2.18 (1.04–4.57)	1.17 (0.79–1.73)	0.044
No anticonvulsant use	1.00	1.00		1.00	1.00	
Anticonvulsant use	5.36 (2.76–10.39)	0.96 (0.53–1.76)	0.000	6.88 (2.91–16.27)	1.19 (0.61–2.33)	0.002

^a^Adjusted for the same confounders as described below Table 2 for any and osteoporotic fracture, but the confounder is not added to the model if it is similar to the drug being investigated

^b^The interaction term (MG × drug use in the previous 6 months) was investigated within the cohort of MG patients and controls

Conversely, within the group of incident MG patients risk of fracture was twofold higher in those with a recent use of antidepressants (AHR 2.15 [95 % CI 1.22–3.79]), twofold higher for anxiolytics (AHR 1.80 [95 % CI 0.97–3.34]) and fivefold increased with recent use of anticonvulsants (AHR 5.36 [95 % CI 2.76–10.39]). Typical osteoporotic fracture risk was threefold higher within incident MG patients with recent use of antidepressants (AHR 3.27 [95 % CI 1.63–6.55]), twofold higher with recent use of anxiolytics (AHR 2.18 [95 % CI 1.04–4.57]) and sevenfold higher with recent use of anticonvulsants (AHR 6.88 [95 % CI 2.91–16.27]). None of the remaining risk factors for fracture, which are described in the “Methods section”, showed a significant increased or decreased risk for any fracture or for fractures at osteoporotic sites. Finally, within the complete cohort with both incident MG patients and control patients, the interaction term between MG and anxiolytics showed statistical significance for osteoporotic fracture (*p* value < 0.05). The interaction term between MG and anticonvulsants showed statistical significance for both osteoporotic and any fracture (*p* value < 0.05).

**Fig. 1:**
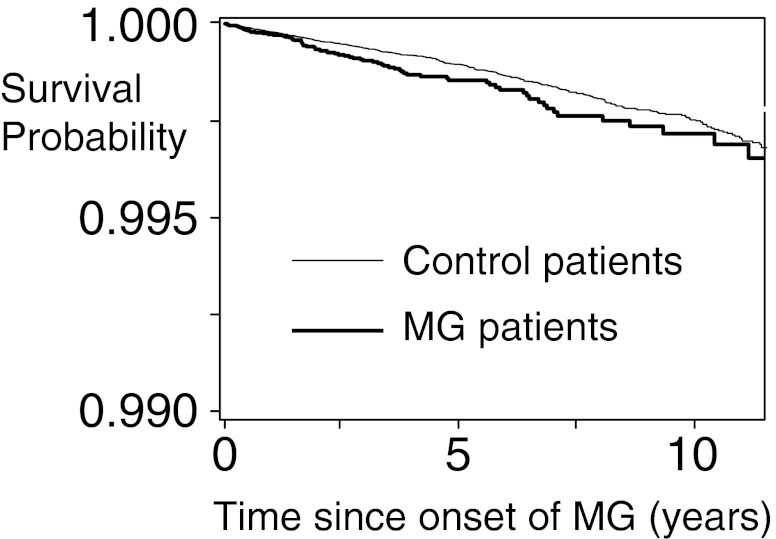
Kaplan–Meier survival curve for any fracture among MG patients versus patients without MG

To further investigate whether a true association between MG and fracture risk had been averaged out by a fluctuating hazard function, we showed that MG duration was not related to fracture risk: 1-year risk of any fracture yielded an AHR of 1.15 (95 % CI 0.88–1.52) in patients with MG versus population-based controls, while 5-year risk (AHRs of 0.97 [95 % CI 0.74–1.28]) and 10-year risk (AHR 0.94 [95 % CI 0.71–1.23]) were not different. The Kaplan–Meier curve as presented in Fig. 1 showed similar results with a non-significant log-rank test (*p* value > 0.05) when MG patients were compared with control patients. In addition, the severity of MG was not related to increased risk of fracture (Table 5). Finally, using MG patients only from the GPRD (without HES data) did not alter the findings.

**Table 5:** Risk of any fracture and fracture at osteoporotic sites in incident MG patients, by severity compared to patients without MG

	Risk of any fracture		Risk of fracture at osteoporotic site	
	Number of fractures	Fully adjusted HR (95 % CI)^a^	Number of fractures	Fully adjusted HR (95 % CI)^a^
MG by severity steps based on pyridostigmine and immunosuppressants use in the 6 months prior^b^				
Grade 1: no use	28	1.00	15	1.00
Grade 2: immunosuppressants only	13	0.67 (0.16–2.80)	6	0.81 (0.13–5.04)
Grade 3: pyridostigmine only	17	0.99 (0.54–1.83)	11	1.14 (0.51–2.54)
Grade 4: both immunosuppressant and pyridostigmine use	17	0.34 (0.07–1.60)	11	0.48 (0.07–3.42)

^a^Adjusted for the same confounders as described below Table 2 for any and osteoporotic fracture, but the confounder is not added to the model if it is similar to the drug being investigated

^b^Immunosuppressants involved are oral glucocorticoids, azathioprine, tacrolimus, cyclosporine, mycophenolate mofetil and methotrexate

## Discussion

Our results show that an incident diagnosis of MG was not associated with a statistically increased risk of fracture or fracture at osteoporotic sites. Further the use of oral glucocorticoids did not alter overall fracture risk, not even when cumulative exposure had exceed >5 g prednisolone equivalents. No association was present between fracture risk and duration or severity of MG. However, MG patients who used CNS medication are at significantly increased risk compared to MG patients without CNS medication.

The most striking finding of this study was that in patients with MG, the use of oral glucocortiods and in particular in high dosages was not associated with an increased risk of fracture. Alternatively, this subgroup of MG patients may have been underpowered, especially the stratification to cumulative high-dose glucocorticoids, with only four reported osteoporotic fractures in the MG population. A different explanation for the lower HRs in MG patients on glucocorticoids, is that pyridostigmine may have anabolic effects, and therefore level out any detrimental effects of glucocorticoids [12, 13]. Cholinesterase inhibitors elevate acetylcholine levels in MG patients [3]. In vitro studies have shown that osteoblasts express acetylcholine receptors, while elevated acetylcholine levels induced osteoblast proliferation [29, 30], which may ultimately result in anabolic effects of bone. In theory, the positive effects of acetylcholine on bone turnover could level out the negative effects of oral glucocorticosteroids on bone, which would explain our findings. Moreover, a recent study performed by Wakata et al. [31] showed that Japanese MG patients who received long-term (8.2 years) high-dose prednisolone therapy (maximum 80–100 mg for 4–6 weeks) had a 50 % reduced osteoporosis rate as compared to the general population. A second explanation for lower HRs in MG patients on glucocorticoids is that generally, patients treated with glucocorticoids are exposed to an inflammatory disease. Subsequently, the disease may increase the risk for fracture itself, like rheumatoid arthritis [32]. This inflammatory compound is generally not present in MG patients, except for some inflammatory cells that may be present in muscle [33]. An alternative explanation is that glucocorticoids may decrease fracture risk associated with the disease, thus cancelling out its adverse effects. A last explanation is that MG patients are often treated on alternate days with glucocorticoids [15]. In theory, this might reduce side effects.

Despite associations of MG with falling [5–7] and with glucocorticoid-induced osteoporosis [8, 9], our findings showed no significantly increased risk of fracture. In contrast, our finding of an increased risk of fracture in users of various classes of CNS drugs is in keeping with previous findings [18–21, 34]. The increased fracture risk may be caused by side effects of CNS medication, such as sedation and dizziness, through an increased risk of falling.[35–37]. Use of antidepressants has been associated with orthostatic hypotension [35] and the use of anticonvulsants can be considered a marker for seizures [38]. Both orthostatic hypotension and seizures are risk factors for falling and subsequently for fracture. In addition, the use of SSRIs has been shown to reduce bone mineral density in humans and negatively affected bone strength in rodents [39, 40] probably due to serotonin tranporter inhibition in osteoblasts. This can ultimately lead to an increased risk of fracture. Finally, reduced bone mineral density has also been observed among users of anticonvulsants through an increase of vitamin D catabolism, resulting in an increased bone resorption [41]. MG patients using anticonvulsants had a significantly higher fracture risk as compared with control patients using anticonvulsants, for which the cause is unknown. MG patients and controls using anticonvulsants were equally distributed when stratified to a confirmed diagnosis of epilepsy in the GPRD database. The same applies for a diagnosis of neurological pain, which makes effect modification unlikely. This finding warrants further research.

Our study has several strengths. It is the first study that investigated the risk of fracture in a substantial number of MG patients, and for whom longitudinal drug exposure data were available. It had a reasonable sample size, comprising 1,066 incident MG patients who met the inclusion criteria. The study was population-based and compared MG patients directly with age–gender-matched control patients from the same general practice in a sample that is represenative for the total UK population. This makes selection bias unlikely. We had the ability to statistically adjust our analyses for well-known risk factors of fracture such as gender, age, BMI, smoking status and occurrence of prior fractures.

Our study had various limitations. We did not have access to neurology records, including lab test results for presence of acetylcholine receptor antibodies, which are a diagnostic tool for MG [1]. Information on the diagnosis of MG patients was therefore limited. For this reason, we determined fracture risk not only among all patients with a MG recording in either GPRD or HES, but also among more probable MG patients with more than one recording of MG only. We could only use variables recorded in the GPRD to assign disease severity and classification of severity of disease could have been improved, if we would have had access to tertiary care data such as plasmapheresis. We did not have data on femoral bone mineral density and no data on history of hip fracture among the parents of patients. Only small numbers of incident MG patients were present in the subgroup analyses. For this reason, these data should be interpreted with care. Moreover, no data were present about vitamin D plasma levels, degree of exercise or longitudinal data on body weight. This could have confounded the observed increased fracture risks in patients using CNS medication.

We showed an absence of fracture risk among MG patients using oral glucocorticoids compared to unexposed MG patients and a lower risk compared to control patients using oral glucocorticosteroids, but we were unable to determine any significant difference. This issue warrants further research. In theory, high-dose prednisolone might exacerbate MG, which could have interfered with the analyses. However, glucocorticoid treatment is regularly started with a low dose, which is gradually increased [14, 15]. This minimizes the risk of an exacerbation.

In conclusion, this study showed that MG was not associated with a statistically significant increased fracture risk, not even among MG patients who received high-dose oral glucocorticoids. This suggests that there is no need to alter current management of MG. In contrast, fracture risk was increased among patients using CNS medication. Therefore, fracture risk assessment may be indicated among patients with MG who have recently used CNS medication. Further investigation should be performed to address the underlying mechanism for the observed absence of an increased fracture risk among MG patients exposed to high-dose oral glucocorticoids.
